# The Role of Theory in Biology: How You Can Be, and Likely Already Are, a Theoretician

**DOI:** 10.1002/ece3.73896

**Published:** 2026-06-21

**Authors:** Samuel M. Scheiner

**Affiliations:** ^1^ Retired, Charlottesville Virginia USA

**Keywords:** biology theory, constitutive theory, methodology, model, theory building

## Abstract

Theory is an inextricable component of the scientific process. Nonetheless, many researchers have an uneasy relationship with theory. This paper provides tools to empower researchers to place their work within a theoretical framework, and to aid them in building and using theories. Theories play a variety of roles. Models are used to test hypotheses and make predictions. Constitutive theories are rule sets or blueprints for building models. Constitutive theories can be arrived at inductively by identifying the rule set behind one or more related models. Once in place, they can be used deductively to develop new models or reveal relationships among seemingly disparate models. Unlike the typical mathematical nature of models, constitutive theories are a set of statements that can be assembled by any researcher. Various personas can be adopted in the processes of developing and using constitutive theories. Personas that focus on novelty (the Advocate and the Explainer) are interested in developing new models or constitutive theories, robustness personas (the Semantician and the Tinkerer) look to strengthen existing ones, while conflict personas (the Instigator and the Mediator) compare and contrast seemingly disparate ones. Through collaborations among quantitative and empirical researchers, and conceptual, synthetic, flexible, and expansive thinking, all researchers can be theoreticians.

## Graphical abstract

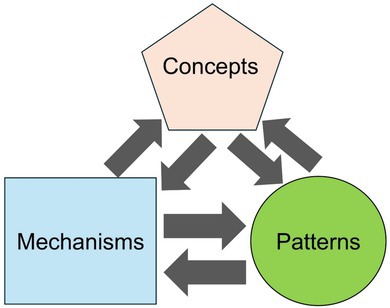

## 1 Introduction

**FIGURE 1:**
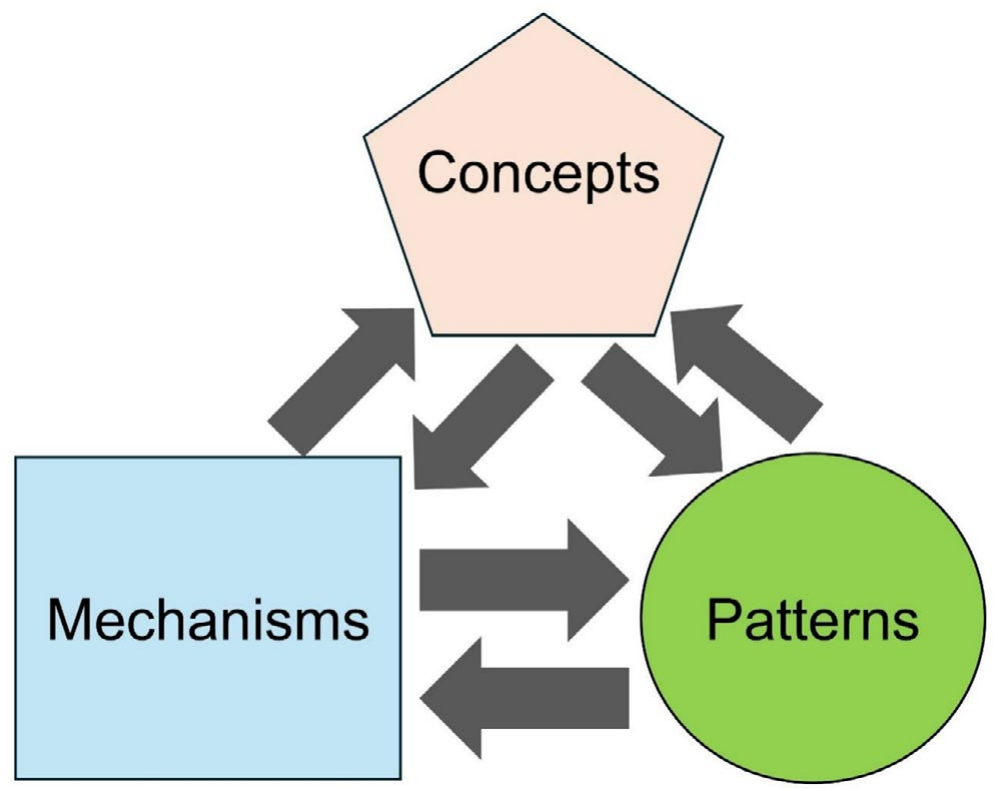
The many types of interactions among observed patterns, proposed mechanisms that might cause those patterns, and the theoretical concepts that explain those mechanisms. The concepts can be organized into constitutive theories that guide model development for testing mechanisms by comparison with empirical patterns. The arrows represent the flow of information rather than causal linkages.

Theory is a vital and inextricable component of the scientific process. Theories transform data into understanding and understanding into predictions. Yet, many researchers have an uneasy relationship with theory, especially with integrating empirical efforts within larger theoretical frameworks that go beyond direct hypothesis tests. The goal of this paper is to empower researchers to situate their work within a theoretical context and aid them in building and using theories. It unites and builds upon two previous efforts, my proposed theory framework (see Types of Theories) and the theory personas (see Types of Persons) of Shaw et al. (2024). Shaw and her collaborators showed how to approach the relationship between mechanisms and patterns (data and models) from different perspectives and framings. In this paper, I add a third component—concepts—to that set of relationships (Figure 1), and explore the relationships between models and more inclusive theories (Table 1).

**TABLE 1:** A schema of theory types that play different roles in the development and use of theory.

Types	Roles
Model	Formalized relationships among entities (quantitative or nonquantitative) that can be compared to data to test hypotheses or to make predictions.
Constitutive theory	A set of propositions that provide a blueprint or set of rules for model building.
General theory	A set of general principles that make explicit background assumptions and create the overall framework for theory development.

A major barrier that prevents empiricists from considering themselves to be theoreticians is that theory is usually equated with mathematical models (Pigliucci 2013). Yet two of the most influential biological theories from the 19th and 20th centuries contain no equations: Darwin's (1859) explication of the theory of evolution by natural selection and Watson and Crick's (1953) proposed structure of DNA and its implications for a replication mechanism. The former is strictly verbal; the latter contains quantitative information about the structure of DNA but is primarily a pictographic and physical model. Although the quantification of biological theories since at least the middle of the 20th century has been important in our ability to more rigorously test our theories, it has also created a barrier to building theories, either due to a lack of quantitative skills in biologists or a misconception that all theoretical work is the crafting of quantitative models.

## 2 Types of Theories

The key to breaching that barrier is to recognize that there are different types of theories, with each type serving a different role. I proposed a theory schema as a means of understanding those types and their roles (Table 1). In this schema, theory is the evolving framework by which we articulate the basis for scientific understanding and expose it to scrutiny and evaluation: it comprises models, constitutive theories, and general theories, as well as associated concepts and confirmed generalities. This schema is aligned with the pragmatic view of theory (Winther 2015).

### 2.1 Models

Models are the theory workhorses where the theoretical rubber meets the empirical road. They are where hypotheses and predictions are proposed and tested. Models are often mathematical, which can make a verbal model explicit and more open to testing. But they need not be quantitative. Examples of nonmathematical models include Watson and Crick's wire model of the structure of DNA, reconstructions of dinosaur skeletons, and mice as model organisms that act as stand‐ins for humans. Models are often also verbal, for example, Darwin's theory of evolution by natural selection (see section 3.2).

### 2.2 Constitutive Theories

Models are embedded within, and derive from, constitutive theories. Constitutive theories are rule sets or blueprints for building models. Often, those rule sets are implicit. Making them explicit can help settle disputes among related models, or at least make points of agreement and disagreement more obvious, as I show in section 3.1. Being explicit about underlying theory makes knowledge advancement more efficient. Learning how to develop and deploy these types of theories is the central goal of this paper. I show how these activities can be accomplished in the absence of quantitative efforts, even in the absence of strong quantitative skills. My own efforts at building constitutive theories are a testament to that last point, as I describe below. It is not quantitative skills that are critical, rather the ability to abstract, synthesize, and generalize.

### 2.3 General Theories

General theories consist of general principles that make explicit the background assumptions and frameworks for constitutive theories and models. I have proposed a set of general theories for biology, consisting of an overarching Theory of Biology, within which are Theories of Cells, Organisms, Genetics, Ecology, and Evolution (Scheiner 2010; Scheiner and Willig 2011; Zamer and Scheiner 2014; Scheiner and Mindell 2020). Those theories are outside the scope of this paper, and I direct the interested reader to those references for more details of their scope, structure, and philosophical bases. Here, I emphasize that my theory framework is more flexible than implied by Table 1. All three types of theory can have domains that are broad or narrow; for example, constitutive theories of evolution due to sexual selection (Kuijper et al. 2012) are contained within the constitutive theory evolution by natural selection (Frank and Fox 2019). The domains of the general theories overlap so that, for example, the constitutive theory of life history evolution (Fox and Scheiner 2019) exists at the intersection of the general theories of Evolution and Ecology.

## 3 The Relationships Between Constitutive Theories and Models

### 3.1 The Inductive Development of a Constitutive Theory From Disparate Models

**FIGURE 2:**
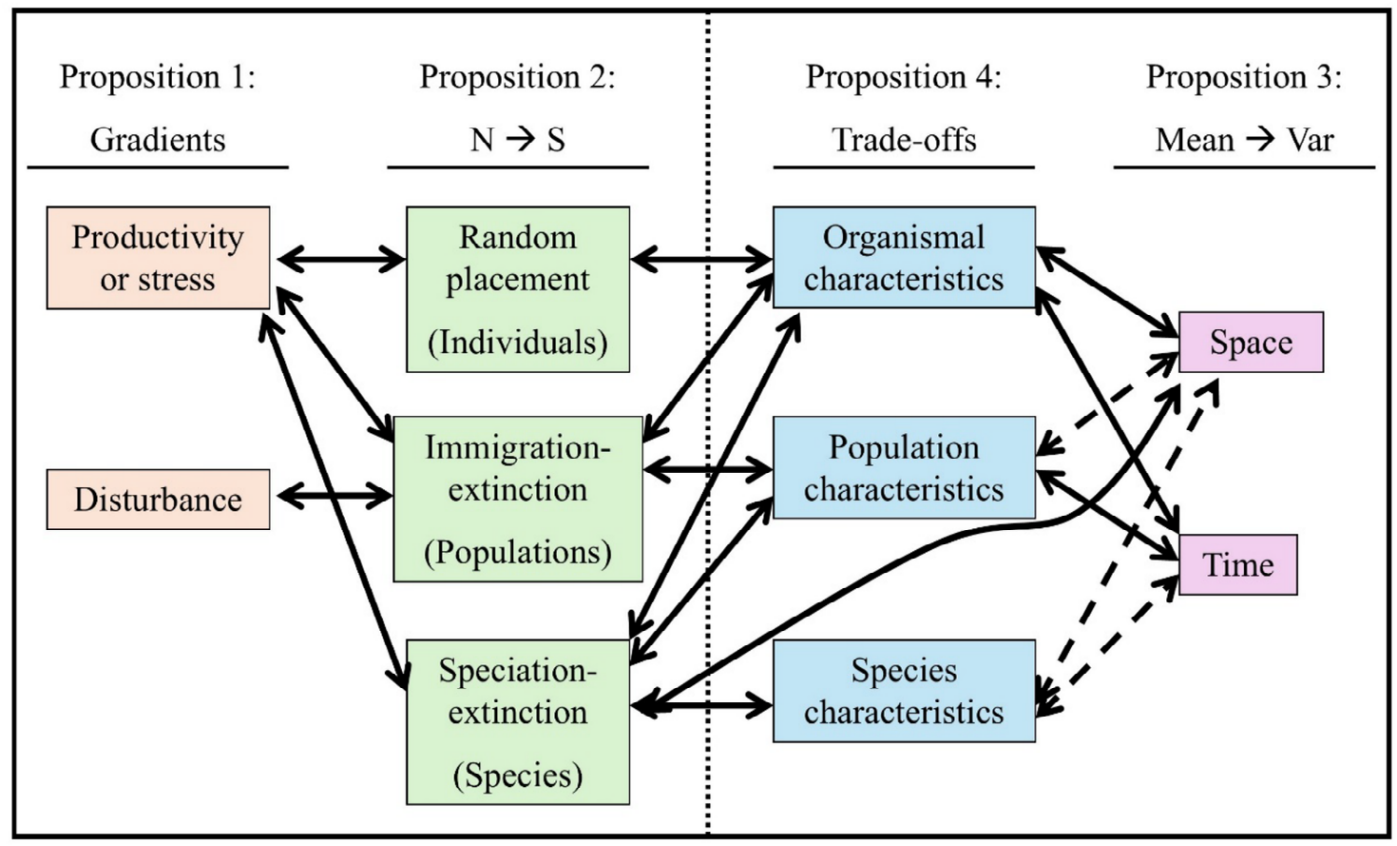
The relationships among the various mechanisms that have been proposed to account for patterns of species richness along ecological gradients. Solid lines indicate linkages that exist in current models. Dashed lines indicate plausible linkages that have not yet been modeled. (Adapted from Fox et al. 2011).

Although constitutive theories provide rule sets for building models, in the absence of an existing constitutive theory, models can provide a roadmap to the development of such a theory. Here, I provide an example of how a seemingly disparate and contradictive set of models can be distilled into a simple constitutive theory. I was part of a working group at the National Center for Ecological Analysis and Synthesis that was examining what, at the time, was a highly debated question: What is the relationship between species richness and productivity (Waide et al. 1999; Mittelbach et al. 2003)? As part of that effort, I was tasked with summarizing and synthesizing the models related to that question. In surveying the literature, I identified 24 papers that proposed models about the causes of patterns of species richness along various types of ecological gradients, not just those related to productivity. A few of those papers had formal mathematical models or were based on computer simulations; most consisted of verbal models. I worked through the stack of papers trying to provide a brief summary of the model in each. My epiphany was the realization that the models in all 24 papers could be summarized in 4 statements or propositions (Table 2), although each of those propositions might be embodied by different processes or mechanisms (Figure 2). For example, the ecological gradient could be due to variation in the amount of productivity or stress, or it could be due to the frequency of disturbance. All models include some version of propositions 1 and 2, whereas propositions 3 and 4 are only used by some. In doing so, I also realized that several of the models, while presented by the authors as being distinct, were actually based on the same propositions and mechanisms, so that the 24 models could be condensed into only 15 (Scheiner and Willig 2005, table 1). In a follow‐up, we further condensed them to just 9 models (Fox et al. 2011, table 13.2).

**TABLE 2:** The propositions that constitute the theory of species richness gradients (Scheiner and Willig 2005; Fox et al. 2011).

A gradient implies one or more limiting resources or conditions that differ in space or time.
2 In a uniform environment of fixed area, more individuals (N) lead to more species (S).
3 Within an area of fixed size or a unit of time of fixed duration, the variance of an environmental factor increases with its mean.
4 All nonmonotonic relationships require a trade‐off in organismal, population, or species characteristics with respect to the environmental gradient.

This process illustrates several aspects of theory development that can be pursued by any researcher, as well as the benefits from such efforts. First, it shows that theory development is often accomplished by identifying the general concepts that encompass particular mechanisms, in this case recognizing that various types of trade‐offs could be gathered under a single header. For example, adapting to using two different resources (Denslow 1980) or balancing growth rate versus competitive ability (Chesson and Huntly 1988) are each trade‐offs of organismal characteristics. Second, it identifies how multiple verbal models that differ in their empirical details are actually the same model (e.g., Denslow 1980; Rosenzweig and Abramsky 1993; Vandermeulen et al. 2001). Third, it delineates which models have all elements (e.g., specific mechanisms) in common and which include different elements. For example, the models of Tilman (1982); Huston and Deangelis (1994), and Leibold (1996) are all models of productivity or stress gradients that involved population‐level immigration‐extinction dynamics and organismal‐level trade‐offs along a spatial gradient. Those models differ from that of Rosenzweig (1995), which employs a temporal gradient, but otherwise contains the same elements. Yet more different are two of the models of Vandermeulen et al. (2001) that involve species‐level speciation‐extinction dynamics and trade‐offs. Fourth, the identification of common elements between verbal and mathematical models suggested ways that verbal models might be quantified. For example, the verbal model of Oksanen et al. (1981) contains all of the conceptual elements that are contained in the analytical model of Wollkind (1976), even though the particular population‐level trade‐offs differ (growth rate versus chaotic dynamics and the intensity of competitive versus predation). Fifth, the diagrammatic presentation of the constitutive theory (Figure 2) identified some combinations of mechanisms that are plausible, but have never been included in the same model, suggesting new directions for model development.

It is possible that more than one constitutive theory can be induced from the same set of models. These alternative could represent competing constitutive theories and we could ask which is better at making rules explicit or capturing the key aspects of all of the models. It might be that both are useful for different purposes (e.g., questions about patterns of biodiversity versus development of conservation strategies). Constitutive theories can be fluid; new models might require changes in current propositions or development of new ones (see next section).

### 3.2 The Deductive Development of Multiple Models From a Constitutive Theory

Deriving models and associated hypotheses from an overarching theory is typically presented as the way that science proceeds. As shown in the previous section, an inductive process that goes in the opposite direction is also possible and probably common. Here, I present a well‐known example of this deductive process to highlight an important potential outcome from the development of constitutive theories.

Darwin's theory of evolution by natural selection can be summarized in various ways; a commonly used one is presented by Endler (1986):

1. If individuals within a population vary in their characteristics.
2. If that phenotypic variation leads to differences in fitness.
3. If that phenotypic variation has a genetic basis.
4. Then, the characteristics of the population will change over generations.

Those four statements form the propositions of a constitutive theory, although alternative formulations are also possible (e.g., Frank and Fox 2019).

This verbal theory led to many different mathematical models of the evolutionary process in the 20th century as part of what is now termed the Modern Synthesis (Smocovitis 1996). Two different mathematical instantiations of the theory are based on seemingly very different genetic concepts. A quantitative genetic concept, which relies on phenotypes as proxies for the underlying genetics is the basis of the breeder's equation: $\Delta \bar{z}=s\sigma_{A}^{2}/\sigma_{P}^{2}$, where $\Delta \bar{z}$ is the change in the mean trait value of a population, *s* is the change in mean phenotype caused by selection prior to reproduction (the strength of selection), $\sigma_{A}^{2}$ is the additive genetic variation in the population, and $\sigma_{P}^{2}$ is the phenotypic variation (Lush 1943; Lynch and Walsh 1998). The variance ratio is often symbolized as $h^{2}$, the heritability of the trait. The form of the breeder's equation shown here has a direct relationship to the constitutive theory with the four elements of the equation corresponding to propositions 4, 2, 3 and 1, respectively, showing how a constitutive theory can act as a rule set for building a model.

An alternative formulation is based on a Mendelian genetic concept of allele frequencies at a single locus: $\mathrm{\Delta p}=\mathrm{sp}{\left(1-p\right)}^{2}/\left(1-s{\left(1-p\right)}^{2}\right)$, where *p* is the population frequency of the allele causing a version of a trait that is under selection, and *s* is the strength of selection, although its meaning is a bit different from that above (Haldane 1924). At first glance, the two mathematical forms seem incompatible, and it was this incompatibility that was part of the debate between the biometricians and Mendelians early in the 20th century (Provine 1971) (I emphasize that my presentation is ahistorical as the breeder's equation was developed after the disagreement was resolved. It serves here as a simple shorthand for a much more complex debate (Radick 2023)). That seeming incompatibility was resolved by Fisher (1918) who showed that the quantitative genetic form could be linked to the Mendelian form by assuming that the trait was determined by many loci, each with a small and additive effect on the phenotype.

Knowing that two seemingly incompatible models derive from the same constitutive theory tells us that they must have some connection. In this instance, the linkage was made by additional assumptions about the genetic basis of trait variation. Making a constitutive theory explicit can suggest that such a linkage can be found and, as in the inductive example above, helps to unify models. Whether such a formalization would have helped to more quickly resolve the debate between the biometricians and Mendelians I leave to the historians.

## 4 Relationships Between Concepts, Mechanisms, and Patterns

One way to give yourself an entry into theory development and usage is to consider the various ways that patterns, mechanisms, and concepts are linked to and influence each other (Figure 1). In my diagram, “patterns” stand for the empirical information that relates to the question or phenomenon of interest. “Mechanisms” are embodied by models that represent the processes that cause those patterns. “Concepts” are the constitutive theories and their components. My diagram is an extension of that of Shaw et al. (2024), see their Figure 1, but it differs in an important regard. Their diagram emphasizes the multiple ways that one or more mechanisms or processes can cause one or more patterns. In their diagram the arrows are unidirectional from mechanism to pattern because they represent causal actions. In my diagram, the arrows represent conceptual linkages. Arrows from patterns to mechanisms or concepts represent inductive reasoning from data to theories, either models or constitutive theories. This flow of ideas can go in various directions. For example, Darwin started with patterns (e.g., differences in finches on islands in the Galapagos) to concepts (the theory of evolution by natural selection), from which others derived various mechanisms (evolutionary models). Many patterns of species richness variation along ecological gradients led to the development of multiple, seemingly disparate, models that we then condensed into a single constitutive theory.

In both instances, the starting point was patterns in data, and that will be the most common pathway for developing theory. However, it is possible for the starting point to be conceptual. For example, Wright's (1932) shifting balance theory of evolution is a constitutive theory that overlaps with the theory of evolution by natural selection, but differs by adding a new component, genetic drift (i.e., changes in gene frequencies in small populations due to sampling effects). The theory did not arise de novo from pure idea, but like all theory had empirical and conceptual forerunners. However, Wright was not trying to explain a particular evolutionary pattern. Rather, he conceived of a novel intersection of processes that could affect evolutionary change. (See Provine (1986) for the history of the development of the shifting balance theory.) Importantly, the previous existence of the constitutive theory of evolution by natural selection created a conceptual framework for Wright's alternative theory.

Thus, constitutive theories can lead to the development of new constitutive theories. They can also suggest where to look for patterns. For example, the theory of evolution by natural selection includes a key new concept: fitness. (For a discussion of definitions of “fitness” see Brommer (2000), Roff (2008), and Orr (2009).) Only after that concept was articulated could researchers determine how to measure it.

## 5 Types of Personas That Can Be Adopted To Be a Theoretician

Shaw et al. (2024) defined and detailed the nature of six personas that can be adopted as a theorist. Their six types are based on two starting points—pattern or mechanism—and three approaches—novelty, robustness, and conflict. I focus on different starting points, the relationship between mechanisms and concepts (models and constitutive theories), and recast their personas (Table 3). Personas that focus on novelty are interested in developing new models or constitutive theories, robustness personas look to strengthen existing ones, and conflict personas compare and contrast seemingly disparate ones.

**TABLE 3:** Overview of six different ways that a researcher can approach the relationship between models and constitutive theories based on the starting point and the approach. For each persona, a typical question is articulated (Adapted from table 1 of Shaw et al. 2024).

Start with	Novelty	Robustness	Conflict
Constitutive theory	Advocate: Here is a new constitutive theory, what models does it lead to?	Semantician: Here is a theory, how are its models related?	Instigator: Here are multiple theories that have overlapping domains, how do their resulting models overlap or relate to each other?
Model	Explainer: Here is a model, what is the rule set that generated it?	Tinkerer: Here is a model, how well does it meet the requirements of its constitutive theory?	Mediator: Here are multiple conflicting models, is there a common rule set or are the models members of different constitutive theories?

### 5.1 The Advocate

The actions of the Advocate are what most researchers would reply if asked to describe what a theoretician does, taking an articulated constitutive theory and shaping particular mathematical models. That is a persona that likely requires substantial quantitative training, although not always. My own work on the evolution of phenotypic plasticity is entirely based on computer simulation models based on “off the shelf” mathematics (e.g., Scheiner 2013).

### 5.2 The Explainer

The actions of the Explainer are the flipside of the Advocate. Ideally, when developing a model, the justifications for the structure and content of a model should be presented in detail. Those justifications can be both mathematical (e.g., time will be treated as continuous versus discrete) and biological (e.g., males have a higher mortality rate than do females). Those justifications can also be abstracted into more general rules that can be used for related models (e.g., individuals have discrete morphologies that lead to different mortality rates, a generalization of differences between males and females).

### 5.3 The Semantician

The role of the Semantician is to be a conciliator. As described above, one of the important milestones in the development of evolution's Modern Synthesis was the reconciliation of the Mendelian and biometrician views of the roles of genes and heredity. That reconciliation was less about one view being wrong and the other right, but that both were based on different aspects of one, always messy, biology. Rarely in biology is it the case that theory resolves to a yes‐this and no‐that dichotomy. Much more often it is like improvisational theater yes‐and, with the question being: What is the relative importance of these multiple mechanisms or processes, all of which occur at least some of the time? The Semantician provides a valuable service by revealing those linkages.

### 5.4 The Tinkerer

Like novelty personas, the actions of the Tinkerer can be a routine part of model development. It is necessary to ensure that a formal model is in alignment with its concepts. But these activities can also work in the other direction. Sometimes, in the process of trying to turn a verbal model into formal mathematical relationships, one discovers problems or limitations of the verbal theory. Perhaps the formal mathematics requires a step (e.g., reproduction) that was not considered by the verbal model. Perhaps quantifying the verbal model leads to a mathematical absurdity (e.g., taking the logarithm of zero). In such cases, the verbal theory needs to be refined, possibly by reconsidering the more general rule set that underlies it.

### 5.5 The Instigator

The role of the Instigator is often as provocateur who brings together or creates clashes between different theories and domains. One of the claims of the proponents of the Extended Evolutionary Synthesis (EES) was that the Modern Synthesis ignored the role of developmental constraints on evolutionary trajectories (Pigliucci and Müller 2010; Laland et al. 2015). (It is beyond the scope of this paper to review this claim; see Smocovitis (2020) and Loison (2025) for an exploration.) The EES proponents were taking on the role of the Instigator, taking models from two apparently disparate parts of biology—development and evolution—that had an overlapping domain—explaining adult form—and asking how those theories related to each other. Formalizing those ideas into constitutive theories might have helped resolve some of the heated disputes over the EES (Laland et al. 2015; Futuyma 2017; Lewens 2019).

For example, in a review of Gilbert and Epel (2009), I extracted a constitutive theory of the evolution of phenotypic novelties from pieces that were scattered throughout the text (Scheiner 2009). The first four propositions of that theory are about the genetic basis of the developmental process. The fifth proposition is about how that genetic basis gets fixed by natural selection, which intersects with the domain of the constitutive theory of the evolution of phenotypic plasticity (Scheiner 2020). The latter theory contains a rich set of models that almost entirely ignore the messy developmental details. Constructing a new constitutive theory that combines components of both might result in new synthetic evolutionary models.

An Instigator can also be a synthesizer by identifying common elements among constitutive theories. For example, the second proposition of the ecological theory of species richness gradients (Table 2) is based on the relationship between area and the number of individuals and species richness. A version of that proposition that relates area and the number of individuals can be found in the theory of evolutionary biogeography (Gillespie et al. 2020). That commonality suggests, perhaps, that those two theories could be united, or maybe a new constitutive theory at the intersection of their domains could be developed.

### 5.6 The Mediator

The Mediator persona is what I adopted in developing the theory of richness gradients (see above). While it may be necessary to have sufficient quantitative training to understand the components of each model, extensive training is not necessary to be able to extract the components of a constitutive theory. In many instances, like my effort, many of the models may not even be quantitative. What is necessary is being able to abstract each model and articulate general concepts.

## 6 How You May Already Be a Theoretician

As emphasized by Shaw et al. (2024), and indicated by my descriptions of the personas, a single individual can adopt more than one, sometimes in the course of a single project or manuscript. Their elucidation here is meant to help you think about the myriad ways that you can interact with models and constitutive theories. Here are a list of activities that you can undertake:

- Examine the models that you frequently work with, and ask: can they be unified?
- Define the domain of the models that you work with.
- Build a constitutive theory for your model(s).
- Make explicit the assumptions of the constitutive theory behind your models.
- Are there other models with the same domain that could be added to your constitutive theory?
- Are there models with overlapping domains that could be added to your constitutive theory with appropriate theory modifications?
- Or, do those other models suggest a different constitutive theory?

It is likely that you are already doing some of this, but have not realized that doing so makes you a theoretician. In particular, you may already have all of the pieces of a constitutive theory for your endeavors, but simply have not formalized them into a set of propositions. Once you start doing so, it becomes easier and easier to make that leap for other topics. I had been thinking in terms of constitutive theories for several years when I was asked to review Gilbert and Epel (2009). Because I was already primed to think in those terms, I was able to extract a constitutive theory that had eluded those authors. The key to such theory development is simply that you be willing to think conceptually and synthetically.

## 7 Conclusions

My goal is to empower you to act as a theoretician by showing how theory is much more than equations. The types of theory activities embodied by the six personas are ones anyone can do. Success in these endeavors can be enhanced by several strategies. First, we need more collaborations quantitative researchers (e.g., mathematicians and statisticians) and empiricists. The pathways between constitutive theories and models involve turning biological knowledge into formal mathematics and confirming that mathematical models match biological reality. Few individuals have the training to do both. Even when the models are computer simulations, empiricists may have some coding ability but not all of the training needed to completely explore the range of possible simulations (as shown by my own collaborations). If constitutive theories are intended to span a variety of empirical systems, no one empiricist may have the knowledge to ensure complete compatibility with a given rule set.

Second, theoretical thinking must be flexible and expansive, not rigid and narrow. Although a vigorous presentation and defense of your own theory is an important part of knowledge advancement, it should not be at the expense of excluding other ideas. It is okay for there to be different constitutive theories and models that provide explanations for the same phenomena. As no theory is ever complete, we often gain understanding through the intersection of different ways of approaching a problem. For example, an evolutionary model can consist of one or a few loci with dominance or epistatic interactions, or be a quantitative genetic model that assumes the existence of many additive loci of small effect, both of which can be mathematical. Or it could be a computer simulation that bridges between the two with a complex genetic architecture that otherwise would be mathematically intractable. In this regard, I echo the advice of Shaw et al. (2024) that calls for flexibility in model development.

Above all, you must be flexible in your own approach to research questions. At times you may focus narrowly on the particular details of your system of study. At other times you need to zoom out and consider how the details of your system might be abstracted to a wider array of situations. This generalization is not the same as simply assuming that the details of your system apply everywhere, or are at least widespread. Even something as fundamental as the genetic code is not universal. Biology is messy. Theoretical thinking and the development of constitutive theories are one way to corral that messiness and advance science.

## Author Contributions

**Samuel M. Scheiner:** conceptualization (equal), formal analysis (equal), investigation (equal), methodology (equal), project administration (equal), resources (equal), validation (equal), visualization (equal), writing – original draft (equal), writing – review and editing (equal).

## Conflicts of Interest

The author declares no conflicts of interest.

## Data Availability

This paper does not include any data.
